# Identification of successful mentoring communities using network-based analysis of mentor–mentee relationships across Nobel laureates

**DOI:** 10.1007/s11192-017-2364-4

**Published:** 2017-03-27

**Authors:** Julia H. Chariker, Yihang Zhang, John R. Pani, Eric C. Rouchka

**Affiliations:** 1 Department of Psychological and Brain Sciences, University of Louisville, Life Sciences Building, Room 317, Louisville, KY 40292 USA; 2 KBRIN Bioinformatics Core, University of Louisville, 522 East Gray Street, Louisville, KY 40202 USA; 3 Department of Computer Engineering and Computer Science, Duthie Center for Engineering, University of Louisville, Room 208, Louisville, KY 40292 USA

**Keywords:** Academic tree, Nobel Prize, Nobel laureate, Nobel network, Academic genealogy, Scientific communities, 91F99, A23

## Abstract

Skills underlying scientific innovation and discovery generally develop within an academic community, often beginning with a graduate mentor’s laboratory. In this paper, a network analysis of doctoral student-dissertation advisor relationships in The Academic Family Tree indicates the pattern of Nobel laureate mentoring relationships is non-random. Nobel laureates had a greater number of Nobel laureate ancestors, descendants, mentees/grandmentees, and local academic family, supporting the notion that assortative processes occur in the selection of mentors and mentees. Subnetworks composed entirely of Nobel laureates extended across as many as four generations. Several successful mentoring communities in high-level science were identified, as measured by number of Nobel laureates within the community. These communities centered on Cambridge University in the latter nineteenth century and Columbia University in the early twentieth century. The current practice of building web-based academic networks, extended to include a wider variety of measures of academic success, would allow for the identification of modern successful scientific communities and should be promoted.

**Electronic supplementary material:**

The online version of this article (doi:10.1007/s11192-017-2364-4) contains supplementary material, which is available to authorized users.

## Background

High achievement in intellectual innovation has been measured in part with the awarding of prestigious honors, such as the Nobel Prize. From the first awards in 1901 through the awards in 2015, a total of 573 prizes have been awarded to 900 laureates, including 875 individuals and 25 organizations (Nobel Prize Facts  2016). Over the years, researchers have attempted to distinguish the characteristics of Nobel Prize winners from those of less eminent scientists in the interest of predicting who will achieve this high-level success in the future (Berry 1981; Zuckerman 1977). To some extent, the scientific knowledge and skill underlying these achievements can be attributed to mentoring. At least one Nobel laureate, Krebs (1967), attributes much of his success in science to his academic mentor. In addition, a variety of studies over the years have focused on the contribution of mentoring relationships to doctoral student success (Allen 2004; Allen et al. 2000; Green and Bauer 1995; Malmgren et al. 2010; Paglis et al. 2006).

In personal interviews with Nobel laureates in the United States, Zuckerman (1977) found that the majority was quite selective in choosing their academic mentor and that many had worked under a Nobel laureate (p. 106). In fact, a number had also collaborated with Nobel Prize winners who were not their mentor. Interestingly, this process generally occurred prior to either individual winning a Nobel Prize (Zuckerman 1967). These Nobel laureates described being just as selective later in their career in choosing students to work in their laboratory. Zuckerman noted that there appeared to be a “pattern of assortative collaboration” based on scientific skill, a concept which is in alignment with current network growth models of preferential attachment (Barabási and Albert 1999; Barabâsi et al. 2002; Price 1976). If this is the case, Nobel laureates should have a greater number of Nobel laureate mentors and students than other scientists, and if these selection tendencies are consistent over generations, Nobel laureates should have a greater number of Nobel laureate ancestors and descendants than other scientists across all academic generations.

Many personal anecdotes by Nobel laureates also describe valuable interaction within the scientific community extending beyond the mentor–mentee relationship (Kohn 1996; Rabi and Code 2006; Rayleigh and Rayleigh 1968; Rigden 2000; Thomson 1937). Laboratories make up a community of researchers in which knowledge and skill is shared within generations between academic siblings. Contact may also occur across multiple generations with grandmentees and with the academic siblings of a mentor. Assortative collaboration in the mentor–mentee selection process should create a situation in which Nobel laureates are surrounded by a higher number of Nobel laureates within a few generations in all directions.

Identifying these academic family patterns would have value in providing evidence that academic lineage and academic community have a role in individual success. Although this seems intuitively correct, and there are certainly personal statements by Nobel laureates paying homage to the role of mentoring in their success, to our knowledge, this has not been quantified. As others have pointed out, Nobel laureate status is certainly not the only measure of academic success, and there are many highly skilled scientists with significant contributions to science who have not been recipients of the Nobel Prize. However, Nobel laureate status provides a direct, easily accessible, measure of success that allows for an initial assessment of these patterns. As other indicators of academic success become easier to access on a large scale, such as number of publications and citations, this line of inquiry could be extended beyond Nobel status and used to identify successful mentoring communities within the realm of high-level science. The observation of these communities would provide valuable clues to creating fertile environments for scientific innovation.

With modern technology and the benefits of crowd-sourcing, generations of mentoring relationships are now represented in networks such as The Academic Family Tree (“Academic Tree”) (David 2016; David and Hayden 2012), greatly facilitating the study of mentoring on a large scale. The Academic Tree is a vast crowd-sourced network containing mentor–mentee relationships across several interconnected domains of science, including all areas of science for which a Nobel Prize is awarded. To study the mentoring relationships of Nobel laureates, we counted the number of Nobel laureate family members for 57,831 individuals in the largest subnetwork of The Academic Tree, restricting the analysis to doctoral student-advisor relationships. This subnetwork included 402 scientists awarded the Nobel Prize in chemistry, physics, and physiology or medicine with one scientist awarded a Nobel Prize in economics and one a Nobel Peace Prize.

We used negative binomial regression analysis to determine whether Nobel laureates had a greater number of Nobel laureate academic family than non-Nobel laureate scientists. In particular, zero-inflated models (Zuur et al. 2009) were used to account for individuals in the network with no ancestors or descendants reported, and thus no possibility of Nobel laureate family members. This also covered individuals in the network who were not alive when they or their colleagues might be eligible for the Nobel Prize. To handle the interdependency inherent in all network data, significance levels were obtained by comparing the outcomes of the analysis to results obtained from many topologically identical networks in which Nobel status was randomly assigned across all individuals in each of the networks.

We assessed family structure in several ways. We examined the number of Nobel laureate ancestors across all generations in the network as well as the number of local and global descendants. Local descendants covered two generations in the network and included mentees and grandmentees, whereas global descendants comprised all generations of descendants. We also identified the number of Nobel laureates within each individual’s local academic family which included three generations in all directions in the network. Three generations encompassed an individual’s mentor, grandmentor, great-grandmentor, mentees, grandmentees, great-grandmentees, siblings, aunts, and uncles.

In all cases, Nobel laureates had a greater number of Nobel laureate family members than non-Nobel laureates. In a visual analysis of the network, clusters of Nobel laureates awarded prizes in the same category could be seen with areas of greater prize diversity where the clusters overlapped. To explore this further, we measured the heterogeneity of prize categories in each individual’s local academic family. Not surprisingly, as the number of Nobel laureates in an academic family increased, so did the diversity of prizes, and this was true for both Nobel laureates and non-Nobel laureates.

This analysis also led to the identification of several historical scientific mentoring communities with high concentrations of Nobel laureates. In some instances, Nobel laureates were directly connected to one another over three and four generations of scientists. Biographical and historical accounts offer the only access to characteristics associated with these successful communities. However, with the expansion of current network databases to include a variety of performance measures for all scientists, new methods could be used to identify modern scientific communities and to study them more directly.

## Methods

### Network collection

The Academic Tree network of mentor–mentee relationships was obtained for analysis from Academic Tree on November 2, 2015 (David 2016; David and Hayden 2012). Academic Tree is a web-based database of academic mentor–mentee relationships that uses a crowd-sourcing method for the collection of information. Individuals can voluntarily provide information regarding academic relationships through the Academic Tree website. Academic Tree can be decomposed into an interconnected set of 68 domain specific networks, and it is possible for an individual to be listed in more than one domain. For example, a Cell Biology Tree exists for individuals working in cell biology, and a Genetics Tree exists for those working in the field of genetics. An individual working in both areas can identify themselves as belonging to both trees.

As can be seen in Table S1 (Online Resource 1), the Academic Tree database holds several types of information, including an individual’s specific research area, major research area (i.e., one or more of the domain specific trees), and five possible academic relationships between individuals in the network, including doctoral student-advisor relationships. The database was received from Academic Tree in SQL format which included an edge file and a node file. There were 114,949 entries in the node file and 260,201 entries in the edge file.

### Network filtering

Several steps were taken to enhance and filter the network prior to analysis. In the original files, there were 484 individuals listed as Nobel laureates. However, 47 additional Nobel Prize winners could be identified in the file and were labeled as such. The Nobel Prize category and year were added for all Nobel laureates. All information regarding Nobel laureates was obtained from the Nobel Foundation (Nobelprize.org 2016). This network was first filtered to include only relationships between doctoral students and advisors (see Online Resource 1, Table S2, Filter 1). Next, because the analysis was focused on Nobel laureate scientists in the realm of biology, chemistry, and physiology or medicine, the network was filtered to included only individuals listed in at least one science tree (Online Resource 1, Table S2, Filter 2). As a result, the majority of Nobel laureates winning prizes for peace, literature, and economics were removed.

In the last step, the strongly connected components within the larger network data set were identified using network analysis tools in Gephi (Bastian et al. 2009). Table S3 in Online Resource 1 lists the number of strongly connected components of different sizes along with the number of nodes and the number of Nobel laureates associated with each component size. The largest strongly connected component of 57,831 nodes was significantly larger than any of the other components and held the vast majority of Nobel laureates (402 of 472). In fact, in Table S4 (Online Resource 1), this was a significant majority of all Nobel laureates in physics (58.2%), physiology or medicine (65.2%), and chemistry (86%). All Nobel laureates in the largest strongly connected component of the network received prizes in chemistry, physics, and physiology or medicine with one exception, Herbert Simon, a highly interdisciplinary scientist who won the Nobel Prize in economics. There were no prize winners in literature, and only one peace prize winner, Linus Pauling, who was also awarded the Nobel Prize in chemistry. Given that 71 percent of all individuals and 85 percent of Nobel laureates were in this large subnetwork, with a good distribution of Nobel Prize winners across chemistry, physics, and physiology or medicine, this subnetwork appeared to be a representative sample of the larger population and suitable for further analysis.

The 70 remaining Nobel laureates, those outside the large subnetwork, were mainly located in small subnetworks of 2–50 individuals. Although it would have been possible to count the number of Nobel laureate family members in these subnetworks and include this in our analysis, we considered it inappropriate given the large amount of missing data that must certainly be associated with these subnetworks. It is well-known that smaller samples are more likely to contain extreme values, providing a less accurate representation of the larger population. As a crowd-sourced network, The Academic Tree is a continuously evolving sample of a much larger population. As new information is added to the network, it is easy to imagine that many of these smaller networks will become connected to the larger subnetwork and can be included in future analyses.

In exploring the largest subnetwork, it was clear that historically documented mentoring relationships had been entered in The Academic Tree for individuals who would not have lived during a time when they would be eligible for the Nobel Prize. This was a concern because this would produce outcomes of zero Nobel laureate family members from a source unrelated to our question of interest. One obvious option would be to remove these individuals from the network, by perhaps pruning the network to extend no further than one “academic lifetime” prior to the first Nobel awards in 1901. However, there are no dates associated with individuals in The Academic Tree other than the year Nobel laureates were awarded the Nobel Prize. The historical accounts of well-known non-Nobel laureate scientists would provide some information regarding the time period these individuals were working, but there were many individuals for whom access to this information was unavailable. A more tenable option was to control for any negative effects in the analysis. Of particular concern was the over inflation of zero outcomes, which zero-inflated regression models are specifically designed to handle. The benefit of these models is discussed in the “Results” section.

All further network visualization and filtering was done in Cytoscape (Kohl et al. 2011). The Cytoscape filtering tool was used to identify and visualize the subnetworks displayed in several figures presented in the paper.

### Data analysis

We initially explored standard forms of network analysis but decided they would not adequately answer our research question. For example, ERGM (exponential random graph models) could give us the probability of a relationship (edge) between two nodes in the network that are Nobel laureates; however, we wanted to look at the effects of this selection process well-beyond the direct relationship between mentor and mentee. Main path analysis would calculate a main path through the network by selecting nodes at each step with the highest number of connections (i.e., degree centrality); however, we simply needed to trace each individual’s ancestral line and descendants. Similarly, we found that methods for community detection were not designed for use with directed acyclic graphs, nor would they necessarily reflect the specific topological structure we were interested in, which in this case was the immediate family members an individual might conceivably come into contact with over the course of an academic career. Fortunately, network search algorithms exist (e.g., breadth and depth first search algorithms) that allowed us to define the topological structure in the network we wanted to measure: a direct path to an individual’s earliest recorded ancestor, all paths to an individual’s last recorded descendants, all paths through two generations of descendants, and all paths to academic family members within a radius of three generations. Consequently, our outcome measure, number of Nobel laureates, actually reflects the number of Nobel laureates within a specific topological structure in the network.

A breadth first search algorithm was used to calculate the number of family members and the number of Nobel laureate family members for each individual. This was instantiated in a custom C++ program which takes a directed acyclic graph as a node and edge list. The node list contains node id, Nobel status, and Nobel Prize category. The edge list contains source and target nodes. The direction in the network (forward, backward, or both) and the number of academic generations (i.e., steps in the network) to be calculated is specified as input to the program. The shortest path between the two most distant nodes in the network (i.e., the network diameter) measured 31 steps and can be thought of as the number of generations in the network. Therefore, number of ancestors/Nobel ancestors was calculated as 31 steps backward from an individual node while number of descendants/Nobel descendants was calculated as 31 steps forward from an individual node. The number of mentees/grandmentees (M/GM) and Nobel mentees/grandmentees was calculated as two steps forward in the network, the first step encompassing all mentees and the second step encompassing all grand mentees. The number of local family members and Nobel local family members was calculated as three steps forward and backward in the network in all directions. Consequently, this included an individual’s mentor, grandmentor, great grandmentor, mentees, grandmentees, and great grandmentees as well as academic aunts, uncles, and cousins. While calculating number of Nobel laureate family members the program also tracks the number of Nobel Prizes in each category.

### Heterogeneity computation

A measure of heterogeneity was used to calculate the diversity of Nobel Prizes awarded within three steps of each individual in the network (Eq. 1) where *L* is the number of Nobel laureates within a specified distance in the network, *N* is the number of prize categories (5 in this case: chemistry, physics, physiology or medicine, economics, and peace), *n*_*i*_ is the number of Nobel laureates in a specific prize category within a specified distance. The number of Nobel laureates in an individual’s local family was an essential factor in the equation and meant that the scores could not be compared across individuals with different numbers of Nobel laureate family members. Therefore, the scores were normalized to fall between 0 and 1 with 1 representing the greatest possible diversity for a given number of Nobel laureates.

1 $$H = \frac{1}{L}*\log_{N} \frac{L!}{{\mathop \prod \nolimits_{i = 1}^{N} n_{i} !}}$$

### Generation of random networks

Hypothesis testing with network data is problematic in that the assumption of independent observations required for many statistical methods is violated, resulting in standard errors that are computed incorrectly (Hanneman and Riddle 2005; Borgatti et al. 2013). Familiar methods of network analysis, such as ERGM, overcome this by comparing the observed data to expected values that are derived from a random probability distribution derived from the data. This allows for more accurate calculation of the significance of coefficients in the model. Although ERGM is dealing with the probability (density) of edges in the graph, we are dealing with counts as our outcome. Therefore, the significance levels in our analyses were adjusted by creating a distribution of expected test statistics, derived from random samples, for comparison with an observed test statistic (Borgatti et al. 2013). This involved permuting values for the predictor variable (Nobel status) with respect to an outcome variable (number of Nobel laureate family members) for one thousand samples, performing the statistical analysis on each of the random samples, and then counting the number of test statistics on the permuted data that were greater than or equal to the observed statistic. This number was then divided by the number of random samples to produce an adjusted *p* value. For example, if three random test statistics of 1000 permuted samples are greater than or equal to the observed test statistics, the *p* value would be adjusted to 0.003.

**Fig. 1:**
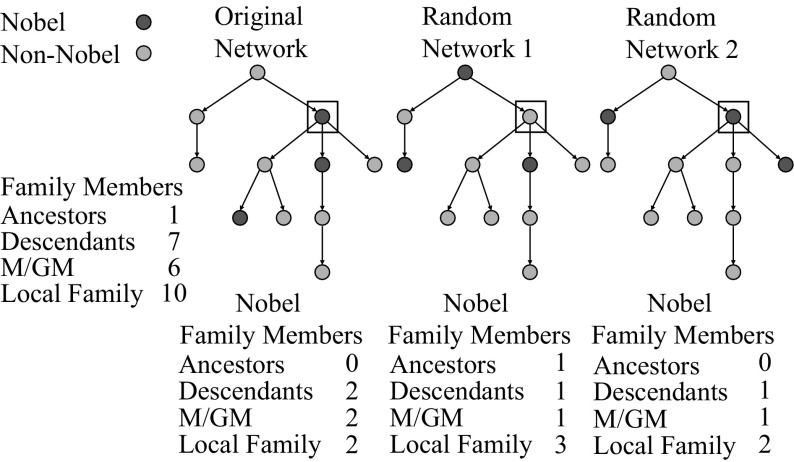
The six largest subnetworks composed entirely of Nobel laureates. *Bold* edges indicate mentoring relationships recorded in The Academic Tree subsequent to the receipt of the dataset

To accomplish this, the C++ program described earlier had options available for generating 1000 networks with Nobel status randomly assigned to nodes across the network in the same proportion as the true data, each time recomputing outcome measures for each node. As can be seen in Fig. 1, this produced alternate networks with equivalent topology (i.e., the same number of family members and academic structure for each node) but randomly distributed Nobel laureates and thus, random outcomes.

## Results

### Descriptive summary

There were 402 Nobel laureates and 57,429 non-Nobel laureates in the largest connected network. Of the Nobel laureates, there were 116 individuals awarded a Nobel Prize in physics, 146 awarded a prize in chemistry, and 137 awarded a prize in physiology or medicine. One individual was awarded a prize in economics, and two individuals were awarded two prizes. Of these, one individual was given a prize in physics and chemistry. The second individual was given a prize in chemistry and awarded a peace prize.

As can be seen in Table 1, the distributions for the number of academic family members and the number of Nobel laureate academic family members are positively skewed on all measures. On some measures, the range was quite large, prompting a closer look at The Academic Tree. For example, the range for number of descendants extended to 2628 for Nobel laureates and 13,620 for non-Nobel laureates. However, academic lineages have been recorded across several centuries, justifying these numbers. For example, Michele Savonarola, a non-Nobel laureate physician scientist, practicing in the fifteenth century, has 13,620 descendants, 73 Nobel descendants, and 0 ancestors in the network. Wilhelm Friedrich Ostwald, a Nobel Prize winner in chemistry (1909), has five ancestors and the highest number of descendants in the Nobel laureate group at 2628. In terms of mentees/grandmentees, it appears that Robert B. Woodward, a Nobel Prize winner in chemistry (1965), has 213 mentees/grandmentees recorded, one of whom is a Nobel laureate. In the non-Nobel laureate group, Gilbert Stork, a Professor of Chemistry Emeritus at Columbia University, has 149 mentees/grandmentees, also with one Nobel laureate among them. The range for number of local academic family was also quite large. Robert B. Woodward has the largest local family with 558 members, 5 of whom are Nobel laureates. Of the non-Nobel laureates, Robert T. Paine, Professor Emeritus of Zoology at The University of Washington has 446 local family members. The distributions for all measures are displayed in Fig. S1 in Online Resource 1.

**Table 1:** The range and median for number of Nobel laureate academic family and total number of academic family across all measures for Nobel laureates (NL; *n* = 402) and non-Nobel laureates (Non-NL; *n* = 57,429)

	Number of NL academic family		Number of academic family		Correlation between academic family and NL academic family
	NL	Non-NL	NL	Non-NL	
	Range (Mdn)	Range (Mdn)	Range (Mdn)	Range (Mdn)	Spearman’s *r*
Ancestors	0–6 (0)	0–8 (0)	0–75 (7.5)	0–131 (9)	0.33***
Descendants	0–21 (0)	0–73 (0)	0–2628 (11)	0–13,620 (0)	0.24***
M/GM	0–8 (0)	0–8 (0)	0–213 (5)	0–149 (0)	0.17***
Local family	0–18 (2)	0–17 (0)	3–558 (31)	3–446 (22)	0.17***

The correlation between number of academic family and number of Nobel laureate academic family for each measure is also displayed. Ancestors refer to individuals moving backward in the directed network, and descendants are all individuals moving forward in the network. M/GM refers to the number of mentees and grandmentees (two generations forward), and local family refers to the number of individuals within three generations forward and backward in the network

*** *p* < 0.0001

One reason a strong positive skew was found for ancestors, descendants, and mentees/grandmentees was due to the nature of network data, where some individuals serve as source nodes without ancestors and other individuals serve as sink nodes without descendants. This increases the number of outcomes measuring zero in the data. In this case, having zero Nobel laureate family members is a result of having zero family members. Alternatively, a number of individuals in the network have ancestors or descendants, but none of them are Nobel laureates. Clearly, there was more than one possible source for zero Nobel laureate family members. In the analysis of ancestors who were Nobel laureates, for example, there were 2890 individuals with no ancestors, and thus no Nobel laureate ancestors. On the other hand, there were 37,608 individuals with ancestors, none of whom were Nobel laureates. Similarly, in the analysis of descendants who were Nobel laureates, there were 40,044 individuals having no descendants. At the same time, there were 16,869 individuals with immediate descendants and 17,197 individuals with mentees/grandmentees, none of whom were Nobel Prize winners.

## Approach to analysis

Zero-inflated regression models were developed for analyzing data with two possible sources for zero outcomes. With this approach, two models are estimated, a zero-inflation model and a count model (Hothorn and Everitt 2014; IDRE 2016). The zero-inflation model is estimated first, using a binomial model to estimate the probability of excess zeros in the data (i.e., a zero outcome due to the absence of family). Once this probability is estimated, the probability for the remaining outcomes is estimated using a Poisson or negative binomial model, whichever is appropriate. In the current paper, zero-inflated models were used to control for excess zeros in estimating the number of Nobel laureate ancestors, descendants, and mentees/grandmentees. There was no need for this in estimating the number of local Nobel laureate family members, because inclusion in a connected network necessarily meant that at least one family connection existed.

For all four analyses, negative binomial models were chosen to adjust for greater than expected dispersion in the data (i.e., a high variance to mean ratio). Spearman’s correlations (see Table 1) indicated that the number of Nobel laureate family members was positively related to the size of the academic family, presenting a potential confound. Therefore, in each case, the size of the academic family was entered along with Nobel status as a predictor of the size of the Nobel laureate academic family. As described in the method, the significance level for each analysis was adjusted by comparing the observed test statistics with a distribution of expected test statistics, derived from 1000 topologically identical networks, each with a random permutation of Nobel status. The regression model coefficients and the distributions of random coefficients used to adjust the significance levels of predictors in the models are available in Table S5 and Fig. S2, respectively, in Online Resource 1.

### Regression model outcomes

Nobel laureates had a greater number of Nobel laureate ancestors than non-Nobel laureates did, suggesting that Nobel laureate mentorship may play a role in the development of future Nobel Prize winners (adjusted *p* = 0.003). However, the number of academic ancestors was not a significant predictor of the number of Nobel ancestors (adjusted *p* = 0.389). Similarly, Nobel laureates had a greater number of Nobel laureate descendants than non-Nobel laureates did (adjusted *p* < 0.001) with number of descendants not significantly predicting number of Nobel laureate descendants (*p* = 0.143).

In contrast to the previous two results, the number of mentees/grandmentees did serve as a significant predictor of number of Nobel laureate mentees/grandmentees (adjusted *p* < 0.001). Still, after controlling for family size, Nobel laureates had a greater number of Nobel laureate mentees and grandmentees than did non-Nobel laureates (adjusted *p* < 0.001). Finally, Nobel laureates also had a greater number of local Nobel Laureates in their academic family than did non-Nobel laureates (adjusted *p* < 0.001). The number of local academic family members did not significantly predict the number of Nobel laureates (adjusted *p* < 0.964).

### Identification of Nobel laureate communities

**Fig. 2:**
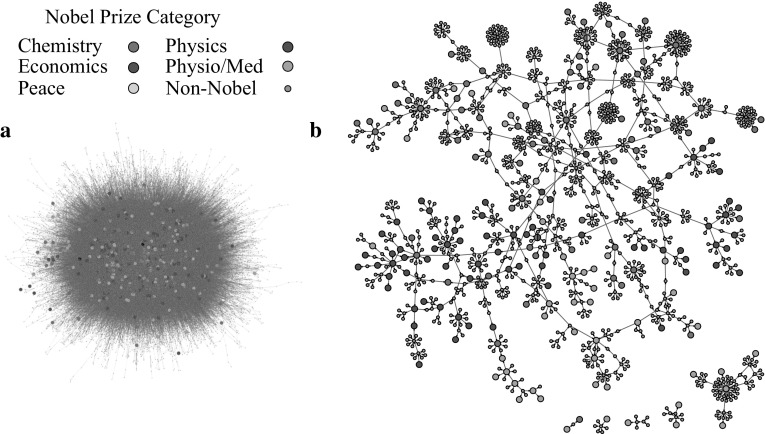
Number of family members and number of Nobel laureate family members computed for an individual network node, *highlighted in the box*, for a directed network (*left*) and two networks in which Nobel status is randomly permuted (*right*)

**Fig. 3:**
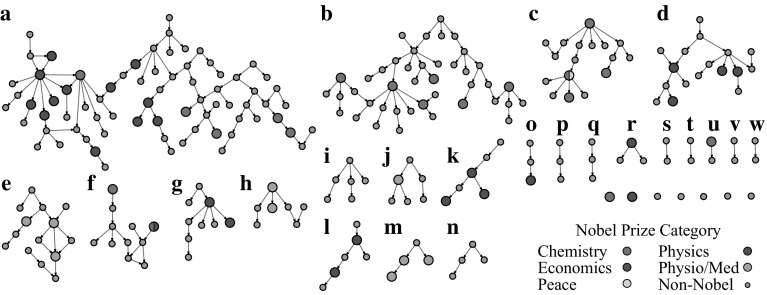
The largest component of The Academic Tree network **a** filtered to include individuals at the 99th percentile for number of Nobel laureate descendants and number of local Nobel laureate family members along with their first neighbors (**b**). The individual names associated with each node in subnetwork** b** are viewable in a high resolution pdf in the supplement (Fig. S3 in Online Resource 1)

To identify highly successful scientific communities, the largest component of The Academic Tree Network, displayed in Fig. 2a, was filtered to include only individuals at or above the 99th percentile for the number of local Nobel laureate family members (99th percentile = 4) and the number of Nobel laureate descendants (99th percentile = 1), along with their first neighbors in the network. This produced one large subnetwork of 1276 individuals and five smaller subnetworks ranging in size from 3 to 68 individuals (Fig. 2b). This network remained quite large, and in Fig. 3, first neighbors were removed, producing a more tractable set of 30 subnetworks for analysis, ranging from 1 to 73 individuals. Nobel laureates in the surrounding academic family who contributed to the scores of these individuals are not pictured. Consequently, these subnetworks only display individuals at the center of the local academic family, making the scale of the two largest subnetworks remarkable. A list of individuals in this group, along with the number of family members and Nobel laureate family members on all measures, is available in Dataset S1 in Online Resource 2. To explore the connectivity among these scientists, high resolution images of Figs. 2b and 3 are available in the supplement with scientist’s names (see Figs. S3 and S4 in Online Resource 1).

The largest subnetwork (Fig. 3a) can be segmented into two early communities by identifying the geographical location of the scientists. One community centered around J. J. Thomson (physics, 1906) and Ernest Rutherford (chemistry, 1908) at Cambridge University, and a second centered around notable scientists such as August Kundt, Wilhem Rontgen (physics, 1901), Johannes Muller, and Hermann von Helmholtz, among others, working across multiple universities in Germany and Switzerland. The German/Swiss community extends to Herman Staudinger (chemistry, 1953) and Leopold Ruzicka (chemistry, 1939) at the right of the subnetwork. Justis Von Liebig, a German chemist considered the founder of organic chemistry (Brock 2002), has the greatest number of Nobel descendants in this group at 53. Eilhard Mitscherlich, Heinrich Magnus, and Johannes Muller follow with 27, 26, and 26 Nobel descendants, respectively. In the Cambridge community, William Hopkins and Edward Routh, well-known non-Nobel laureate mentors (Warwick 2003), lead with 22 Nobel descendants. Their mentees/grandmentees, J. J. Thomson and Ernest Rutherford, both Nobel laureates, have 16 local Nobel laureate family members. David Shoenberg, a British physicist with 17 Nobel laureate family members connects these two major communities.

Interestingly, two additional communities in the largest subnetwork were established in the United States through mentors trained in Germany. Nobel laureate Isador Isaac Rabi (physics, 1944) with eight Nobel descendants, six of which are mentees/grandmentees, serves as the center of one group at Columbia University. William Giauque (chemistry, 1949) and Willard Libby (chemistry, 1960) are at the center of a second community at the University of California, Berkeley.

A significant portion of the second largest subnetwork (Fig. 3b), also contains individuals operating across universities in Germany, and once again, individuals trained in Germany began new communities at universities in Britain, through William Perkins, and universities in the Northeastern United States, through Ira Remsen. In this subnetwork, Friedrich Wohler, a German chemist, has the highest number of Nobel descendants at 39. Johannes Wislicenus, a German chemist, and William Perkin, an English chemist, have the highest number of local Nobel laureate family members at 13. Approximately half of Wislicenus’s Nobel laureate family members are mentees/grandmentees.

Of particular note in the smaller subnetworks is Enrico Fermi (physics, 1938; Fig. 3k) with 18 Nobel laureate family members and six Nobel laureate mentees/grandmentees. Fermi trained and spent his early years as an academic in Italy during the early twentieth century but traveled to Germany to study with Max Born (physics, 1954) and to the Netherlands to study with Paul Ehrenfest (Segre and Hoerlin 2016). Eventually, near the beginning of WWII, and on winning his Nobel Prize, Fermi moved to the United States and joined the Columbia University community centered on Isador Isaac Rabi. In another subnetwork operating around the same time period (Fig. 3d), Max Born, Werner Heisenberg (physics, 1932), Hans Bethe (physics, 1967), and Robert Oppenheimer, among others, can be found. In this network, Arnold Sommerfeld, a non-Nobel laureate mentor to Heisenberg and Bethe, has 16 Nobel laureate family members and 11 Nobel descendants.

### Nobel laureate subnetworks

**Fig. 4:**
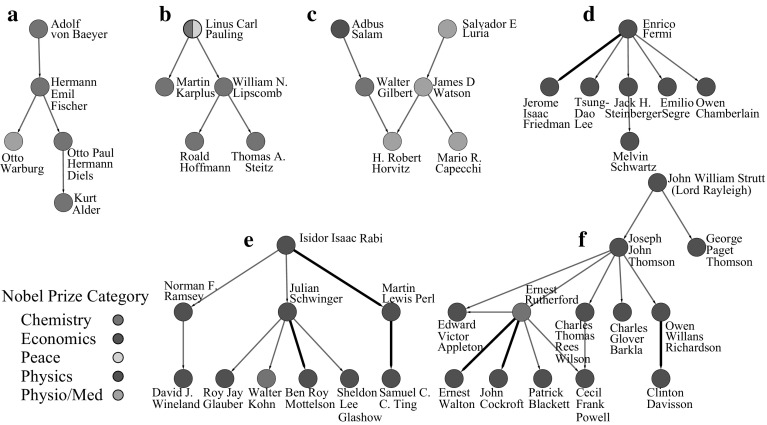
The largest component of The Academic Tree network filtered to include only individuals at the 99th percentile for number of Nobel laureate descendants and number of local Nobel laureate family members. The individual names, the number of local Nobel laureate family members, and the number of Nobel descendants associated with each node are viewable in a high resolution pdf in the supplement (Fig. S4 in Online Resource 1)

On close inspection of Fig. 2b, small subnetworks can be identified that are comprised entirely of Nobel laureates. To explore this further, all non-Nobel laureates were removed from the large strongly connected network (Fig. 2a). Of the 402 Nobel laureates, 260 had no direct connection to another Nobel laureate. However, there were 142 Nobel laureates in 55 subnetworks ranging in size from 2 to 10 individuals. Figure 4 displays the six largest of the subnetworks. An investigation of relationships in these subnetworks identified seven additional connections recorded in Academic Tree after receiving the data used in the analysis. These are indicated by bold edges in the figure. Once again, the scientific communities at Cambridge and Columbia are identified as exceptional with 13 Nobel laureates connected over four generations at Cambridge (Fig. 4f) and 10 Nobel laureates connected over three generation at Columbia (Fig. 4e).

### Heterogeneity of local academic family

In Fig. 2b, clusters of Nobel laureates who were awarded prizes in the same category can be seen with areas of greater diversity appearing where the clusters overlap. In an effort to characterize diversity in the network around Nobel Prize winners, the heterogeneity of Nobel Prize categories was measured within each individual’s local family (see “Method”).

The vast majority of individuals in the network had one or fewer Nobel laureates in their local family. Therefore, the analysis was restricted to individuals with two or more Nobel laureates in the family and where some opportunity for diversity existed (205 of 402 Nobel laureates; 3647 of 57,429 non-Nobel laureates). As the number of Nobel laureates increased, heterogeneity scores also increased (*r* = 0.25, *p* < 0.0001). The distribution of scores for Nobel Prize winners in individual categories was positively skewed (chemistry: 0–0.37, Mdn = 0; physics 0–0.42, Mdn = 0.17; physiology or medicine: 0–0.44, Mdn = 0) and reflective of generally homogeneous clusters of Nobel laureates with some diversity where clusters overlap. There were six Nobel laureates (3 physics, 2 physiology or medicine, 1 physics/chemistry) and 39 non-Nobel laureates with scores at or above the 99th percentile (0.37). Archibald Hill, a Nobel laureate in physiology and medicine (1922; Fig. 3e), scored the highest family heterogeneity (0.44) with four Nobel laureate family members in physiology/medicine, four in physics, and one in chemistry.

These findings prompted us to ask whether there was any difference in family diversity for Nobel laureates and non-Nobel laureates, and the data were fit to a quasibinomial model with Nobel status and number of local Nobel laureate family members as predictors. In the final analysis, heterogeneity scores were not predicted by Nobel status (adjusted *p* = 0.347) after controlling for the number of Nobel laureates in the local family (adjusted *p* = 0.177).

## Discussion

Remarkable connectedness among Nobel laureates is found through generations of mentoring relationships in The Academic Tree network, supporting the notion that assortative processes are at work in mentor–mentee selection. Nobel laureates have more Nobel laureate ancestors, more local and global descendants, and more local academic family members than do non-Nobel laureates. A variety of explanations for this connectedness exist. Nobel laureates undoubtedly possess superior knowledge and skill that individuals in the local academic family, and the greater community, may acquire through a variety of means. Other factors related to the availability of resources and the attraction of talent are no doubt significant contributors to the connectedness of this group. These additional factors are difficult to separate from the transfer of knowledge through mentoring but are an integral part of any successful scientific community and should be valued as such.

Several areas of the network, representing mentoring relationships in historical scientific communities, were identified with high concentrations of Nobel laureates. In some locations, direct connections between Nobel laureates occurred over three and four generations. When exploring biographical and historical accounts of these communities, it was apparent that much greater interconnectedness existed among scientific communities than is reflected by doctoral mentor–mentee relationships. A high degree of interaction occurred throughout these communities. For, example, after completing a dissertation at Columbia University, Isador Isaac Rabi (physics, 1944) spent over a year in Europe where he encountered some of the greatest minds in science, many of whom went on to become Nobel laureates (Rabi and Code 2006). Rabi returned to Columbia to eventually lead the Physics Department and to become a central figure in one of the two most successful communities identified in this analysis (Cole 2009; Cropper 2001; Rigden 2000). In future work, extending the analysis to include a greater variety of mentoring relationships would better capture the true interconnectivity among scientists.

It is significant that many of the successful communities identified by this analysis existed at a time when travel and communication were much more difficult than they are today. Ernest Rutherford (chemistry, 1908) traveled from New Zealand to attend Cambridge as one of the first students admitted from outside the University (Reeves 2008; Thomson 1937). This occurred in the latter half of the nineteenth century prior to the invention of the airplane and intercontinental telephone service. At this point in history, physical proximity was critical to the transmission of ideas and expertise. In modern science, however, virtual meetings, video lectures, online courses, and online databases (e.g., PubMed 2016; Google Scholar 2016) provide remarkably easy access to current, innovative ideas in science. It seems likely that the mentoring patterns among scientists are being radically altered by greater accessibility to information and each other. Still, for many scientists, it is difficult to imagine that virtual proximity could ever be a satisfying replacement for the day-to-day personal interaction found in a positive mentoring relationship.

Biographical and historical accounts provided the sole access to more detailed information about the communities identified in this study. Warwick (2003) offers a particularly valuable and compelling account of the scientific community identified at Cambridge in the latter part of the nineteenth century. However, modern scientific communities could be studied if the types of data required to identify them were available. Although the number of Nobel laureates within an academic community serves as a legitimate measure of success, especially when the research focus is restricted to high-level science, it presents a significant limitation to accurately understanding the effect of assortative processes in mentoring given the high number of eminent non-Nobel laureate scientists. Much more could be accomplished if a variety of other performance measures were readily available. Reliably accurate information regarding publications, impact factors, citations, funding sources, and other awards, would allow for a more sensitive evaluation of success within a community. This could be achieved by a committed effort in the scientific community to collect performance measures from all individuals and universities and to make them available in an open-source database, something The Academic Tree is currently attempting to accomplish.

Several factors would be critical to the success of this endeavor. Primarily, a comprehensive list of all researchers’ publications would need to be available in a centralized, open-source database. Currently, no one source is guaranteed to have a complete set of publications for an individual author (Falagas et al. 2008), and publication information must be obtained from multiple sources, such as Web of Science (2016), Scopus (2016), and PubMed (2016). Furthermore, some of these sources are proprietary and require a fee for use. Google Scholar (2016) has access to several proprietary sources through licensing agreements but does not allow automated searches of its website, something that is a requirement when conducting an analysis of “big data”. As an example, the largest component of the Academic Tree Network analyzed in this study contained 57,831 individuals, making manual search costly in terms of time.

Making a wide variety of performance measures accessible would also increase the value of a database for evaluating scientific success. Number of publications, a measure of productivity, is not a sufficient measure of success. Rather, number of citations, considered a measure of quality, is often factored alongside number of publications in calculations such as the *h*-index (Hirsch 2005). Number of citations is not consistently available in the sources mentioned earlier, and it is not clear how often this information is updated. Along these lines, additional quality measures, such as a journal’s impact factor at the time of an article’s publication, author funding, and additional awards, would be useful in developing new algorithms for measuring the quality of research and the impact of an individual’s and a community’s contribution to science.

Another critical element in developing an effective database involves the assignment of unique identifiers for scientists. This is especially important when dealing with crowd-sourced data. On one hand, crowd-sourcing allows for the collection of data that would be difficult or impossible to obtain otherwise. On the other hand, a quick glance at the Academic Tree dataset makes it clear that ensuring consistency, completeness, and accuracy of the data requires a rigid collection protocol. For example, in the Academic Tree dataset individuals may use all uppercase letters or put a nickname in parentheses, all of which create problems for automated analysis. A unique numerical identifier would allow for much less variation. This problem is clear to many in the scientific community, and it is being pursued by projects such as ORCID (2016). However, to facilitate performance analyses, its use must be required, especially in the authorship section of papers, so that the publications for authors with the same name can be easily distinguished in an automated fashion.

## Conclusion

Using methods of network analysis, Nobel laureates were identified as a highly connected group in The Academic Family Tree network. Several successful mentoring communities could be identified using the number of Nobel laureates as a measure of scientific success. A variety of performance measures exist that would increase the sensitivity of these types of analyses and would allow for the exploration of a greater variety of questions if the measures were collected and made available in a single database. This could provide valuable information regarding individual, institutional, and national factors associated with success in modern science and lead to a greater understanding of best practices. The rewards in such an endeavor would be large, especially in the current climate where there is an increased focus on effective collaboration and teamwork.

## Electronic supplementary material

Below is the link to the electronic supplementary material.

Supplementary material 1 (PDF 4964 kb)

Supplementary material 2 (XLSX 43 kb)
