# Supplementary material for: Identification of successful mentoring communities using network-based analysis of mentor–mentee relationships across Nobel laureates
Source: Scientometrics. 2017 Mar 27;111(3):1733–49. doi: 10.1007/s11192-017-2364-4 (PMC5438421; doi:10.1007/s11192-017-2364-4)
Supplement: Supplementary file 1 — Supplementary material 1 (PDF 4964 kb) [file 11192_2017_2364_MOESM1_ESM.pdf]

Supplemental Information

Julia H. Chariker, Yihang Zhang, John R. Pani, & Eric C. Rouchka*. Identification of Successful Mentoring Communities using Network-based Analysis of Mentor-Mentee Relationships across Nobel Laureates, *Scientometrics*.

* Corresponding Author: Department of Computer Engineering and Computer Science, Duthie Center for Engineering, Room 208, University of Louisville, Louisville, KY, 40292, USA; eric.rouchka@louisville.edu

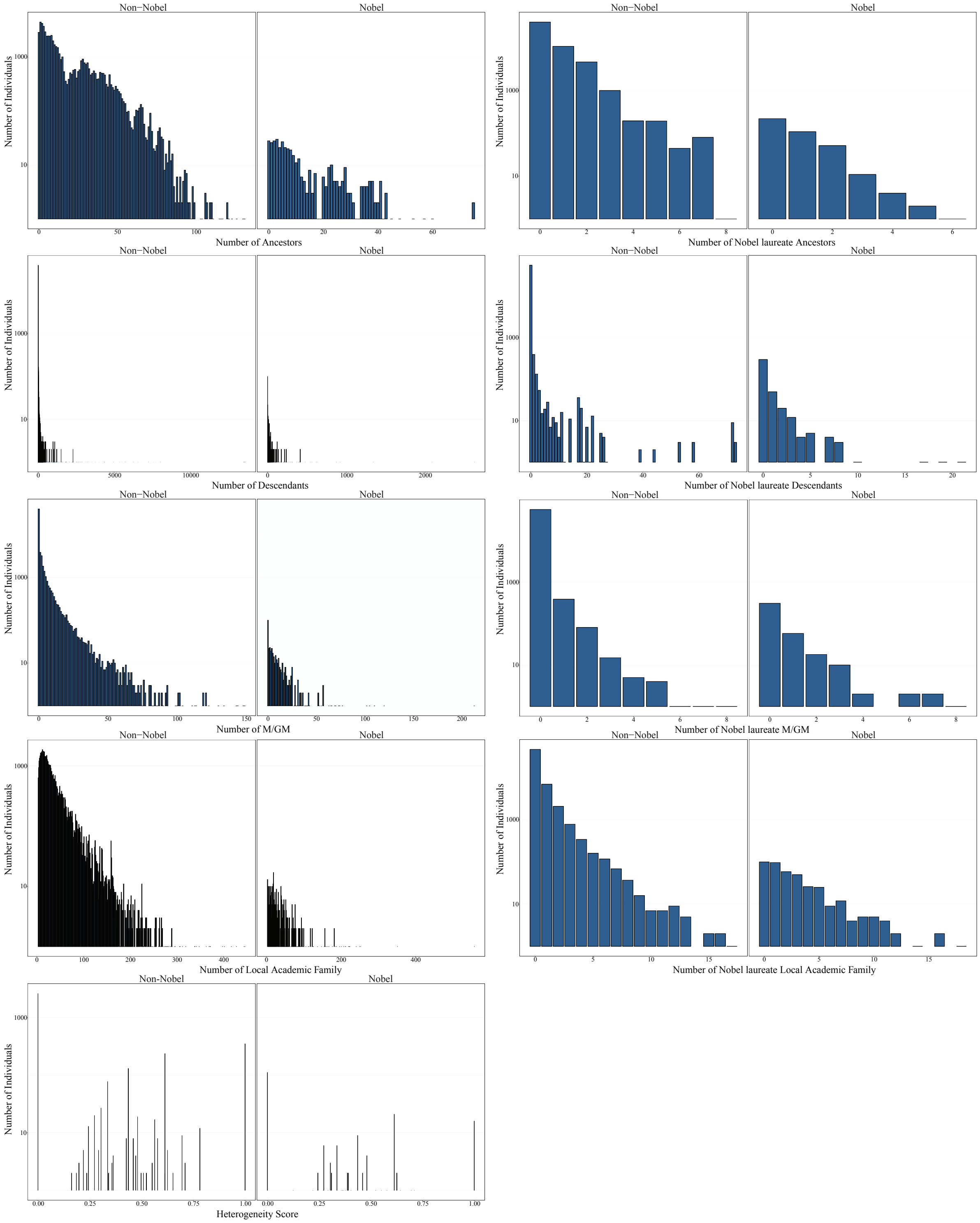

Fig. S1. Frequency distributions for Nobel laureates and non-Nobel laureates for number of ancestors, Nobel laureate ancestors, descendants, Nobel laureate descendants, mentees/grandmentees (M/GM), Nobel laureate M/GM, local academic family members, Nobel laureate local academic family members, and heterogeneity. The Y axis is on a log scale.

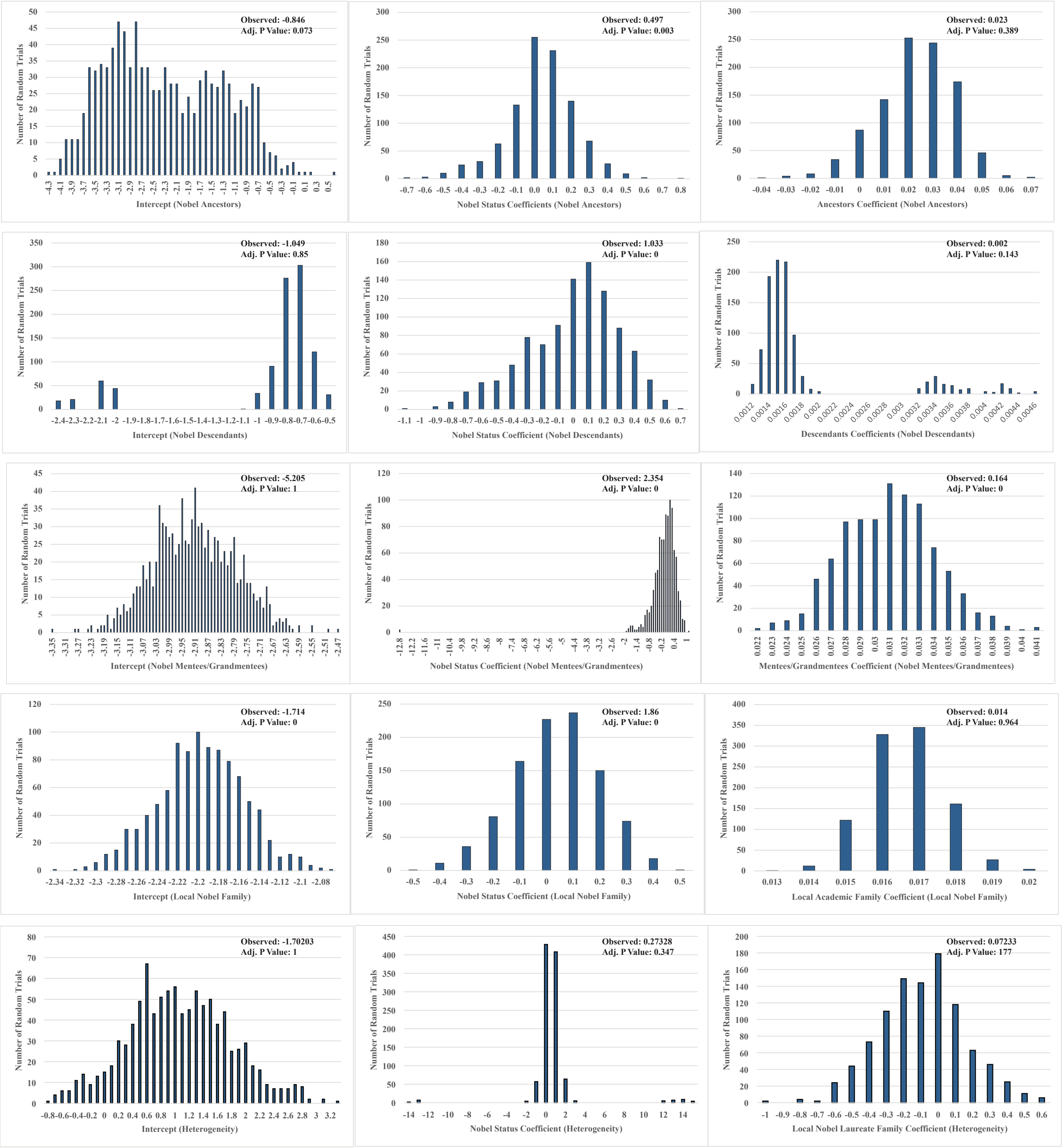

Fig. S2. Frequency distributions for the random intercept, the random Nobel status coeffiecient, and the random family size coefficient generated from 1000 random permutations of Nobel status for number of Nobel ancestors (row 1), number of Nobel descendants (row 2), number of Nobel mentees/grandmentees (M/GM, row 3), and number of local Nobel academic family members (row 4). Frequency distributions for random intercept, random Nobel status coefficient, and random local Nobel academic family coefficients generated for 1000 permutations of Nobel status for the heterogenetity measure is displayed in row 5.

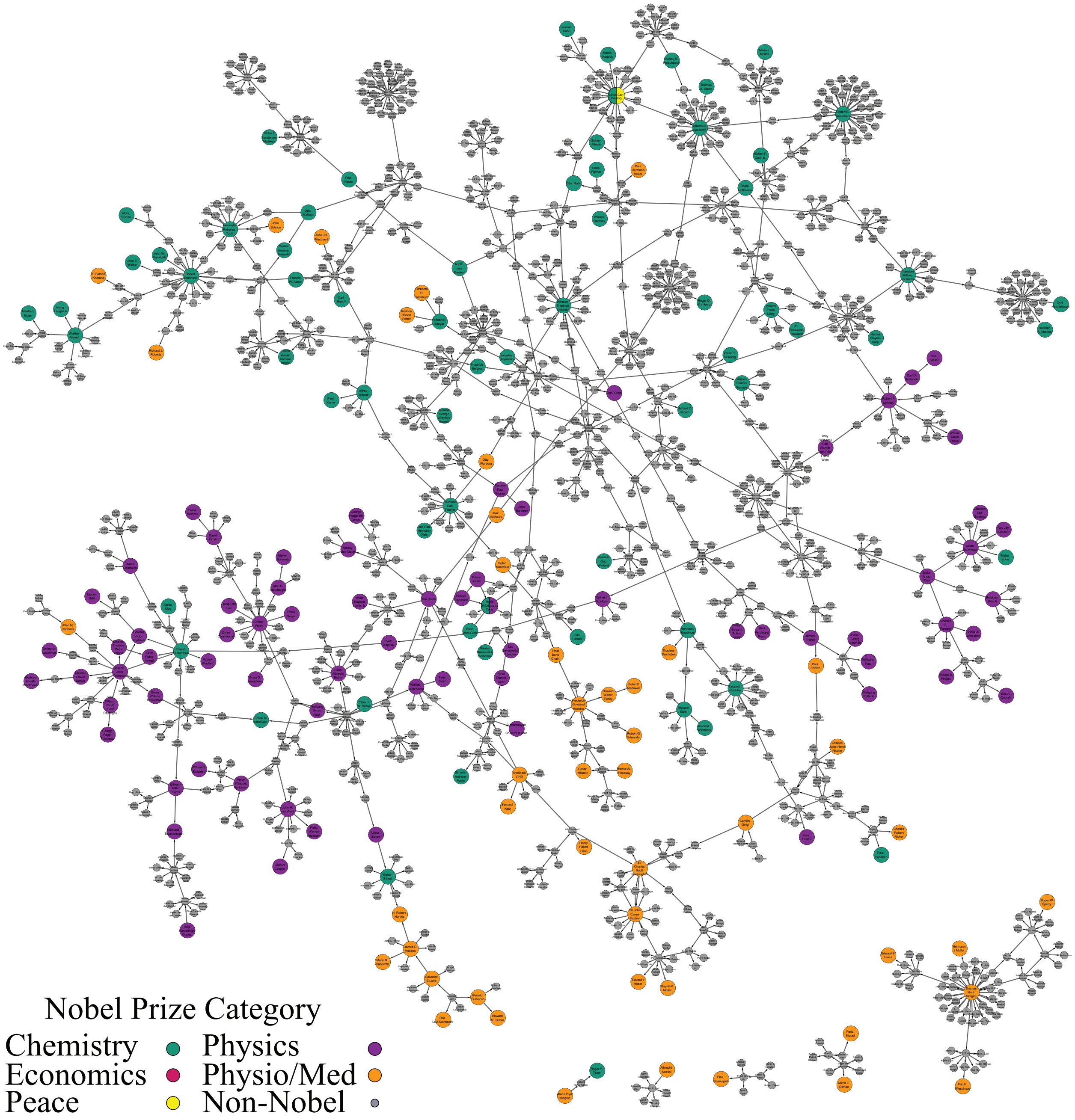

Fig. S3. The largest component of The Academic Tree filtered to include individuals at the 99th percentile for number of Nobel laureate descendants and number of local Nobel family members along with their first neighbors. Individual names are available on zooming.

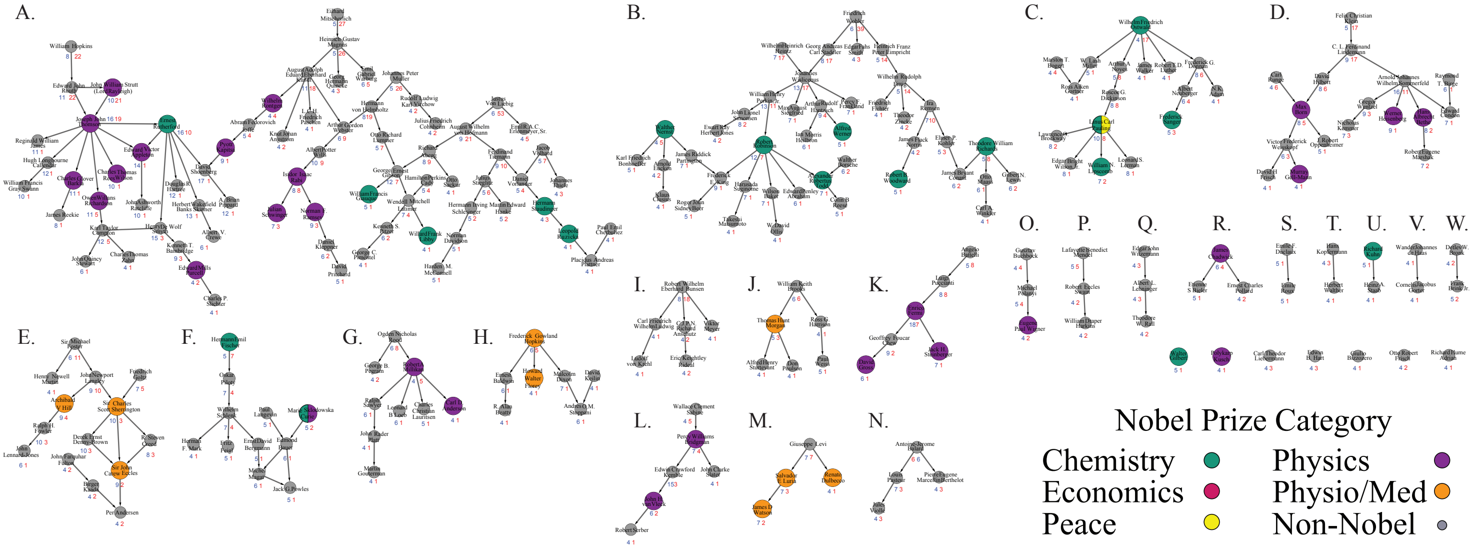

Fig. S4. The largest component of The Academic Tree network filtered to include only individuals at the 99th percentile for number of Nobel laureate descendants and number of local Nobel family members. Individual names, number of local Nobel family (blue), and number of Nobel descendants (red) are available on zooming.

Table S1. A description of information available in the Academic Tree Network.

Node File	Edge File
Column 1: Person Id.	Column 1: Edge Id.
Column 2: First Name	Column 2 Student (Target Node)
Column 3: Middle Name	Column 3: Mentor (Source Node)
Column 4: Last Name	Column 4: Relationship
Column 5: Degree	0 - Research Assistant
Column 6: University	1 - Graduate Student
Column 7: Research Area	2 - Postdoc
Column 8 Major Research Area	3 - Research Scientist
Column 9: Award	4 - Collaborator

Table S2. The number of non-Nobel laureates, Nobel Laureates, Nobel laureates in different prize categories, and number of connections for each data set in the data filtering process.

Category	Doctoral Relationships Only (Filter 1)	Individuals In At Least One Science Tree (Filter 2)	Individuals In Largest Connected Subnetwork (Filter 3)
Individuals (Nodes)	91,608	81,000	57,831
Non-Nobel	91,127	80,528	57,429
Nobel	481	472	402
Physics	133	133	116
Chemistry	159	159	146
Physiology or Medicine	172	172	137
Peace	4	2	0
Literature	2	1	0
Economics	9	3	1
Physics/Chemistry	1	1	1
Chemistry/Peace	1	1	1
Connections (Edges)	85,325	80,731	61,849
Number of Major Areas	66	60	57
Major Areas	Advertising,Alzheimers, Anatomy,Animal Science, Anthropology,Applied Physics, Batten Disease,Baylor College of Medicine,Animal Behavior, Biomechanics,Biomedical Engineering, Bronchopulmonology,Cell Biology,Cell & Gene Therapy, Chemistry,Computational Biology,Computer Science, Crystallography,Communication Sciences and Disorders, Development,ear,Economics, Education,Epidemiology, Engineering,Evolution, Fluid Dynamics and Combustion,Fly,Genetics, Geography,History,Hypnosis, Infectious Disease,Law, Linguistics,Literature, Marine Ecology,Math, University of Michigan, Microbiology,Music,Music Therapy,Mycology, Neuroscience,Neuro-oncology, Neuropathology,Oceanography, Organizational Communication, Pediatric Surgery,Philosophy, Physics,Physiology,Plantsys, Political Science,Fission Yeast Genetics,Primatology, Experimental Psychology, Sociology,Society for the Study of Ingestive Behavior,Science and Technology Studies, Telomere and Telomerase Research,Terrestrial Ecology, Theoretical Ecology,Theology and Biblical Studies, Writing Studies	Advertising,Alzheimers, Anatomy,Anthropology, Batten Disease,Baylor College of Medicine,Animal Behavior, Biomechanics,Biomedical Engineering, Bronchopulmonology, Cell Biology,Cell & Gene Therapy,Chemistry, Computational Biology, Computer Science, Crystallography, Communication Sciences and Disorders,Development,Ear, Economics,Education, Epidemiology,Engineering, Evolution,Fluid Dynamics and Combustion,Fly,Genetics, Geography,History,Hypnosis, Infectious Disease,Law, Linguistics,Marine Ecology, Math,University of Michigan, Microbiology,Music,Music Therapy,Mycology, Neuroscience,Neuro-oncology, Neuropathology,Oceanography, Philosophy,Physics,Physiology, Plantsys,Fission Yeast Genetics,Primatology, Experimental Psychology, Sociology,Society for the Study of Ingestive Behavior,Science and Technology Studies, Telomere and Telomerase Research,Terrestrial Ecology, Theoretical Ecology,Theology and Biblical Studies, Writing Studies	Advertising,Alzheimers, Anatomy,Anthropology, Baylor College of Medicine, Animal Behavior, Biomechanics, Biomedical Engineering, Cell Biology,Cell & Gene Therapy,Chemistry, Computational Biology, Computer Science, Chrystallography, Communication Sciences and Disorders,Development,Ear, Economics,Education, Epidemiology,Engineering, Evolution,Fluid Dynamics and Combustion,Fly,Genetics, Geography,History,Hypnosis, Infectious Disease,Law, Linguistics,Marine Ecology, Math,University of Michigan, Microbiology,Music,Mycology, Neuroscience,Neuro-oncology, Neuropathology,Oceanography, Philosophy,Physics,Physiology, Plantsys,Fission Yeast Genetics, Primatology,Experimental Psychology,Sociology, Society for the Study of Ingestive Behavior,Science and Technology Studies, Telomere and Telomerase Research,Terrestrial Ecology, Theoretical Ecology,Theology and Biblical Studies,Writing Studies

Table S3. The number of subnetworks of different sizes identified in the filtered data set along with the number of associated nodes and number of Nobel laureates at each subnetwork size.

Subnetwork Size	Number of Subnetworks	Number of Nodes	Number of Nobel laureates
57,831	1	57,831	402
1,441	1	1,441	0
836	1	836	1
51-100	7	416	0
26-50	46	1,451	8
2-25	4,103	18,708	59
1	317	317	2
Total	4,476	81,000	472

Table S4. The number and percentage of all Nobel laureates found in the network and the largest subnetwork.

Category	Number of Nobel Laureates	Number (Percentage) of All Nobel Laureates in Data Set (Filter 2)	Number (Percentage) of All Nobel Laureates in Largest Subnetwork (Filter 3)
Physics	201	134 (66.7)	117 (58.2)
Physiology	210	172 (81.9)	137 (65.2)
Chemistry	172	161 (93.6)	148 (86.0)
Economics	76	3 (3.9)	1 (1.3)
Peace	103	3 (2.9)	1 (1.0)
Literature	112	1 (0.9)	0 (0.0)

Table S5. Model predictors, estimated coefficients, standard errors and significance estimates for each outcome measure evaluated.

Outcome	Predictor	Estimate	S.E.	z value	Pr(>|z|)	Number Random>Obs.	Adj. P Value
		Count model coefficients (negative binomial with log link)*					
Nobel Ancestors	Intercept	-0.846	0.027	-30.850	< 2e-16	73	0.073
	Nobel Status	0.497	0.072	6.913	4.74E-12	3	0.003
	Ancestors	0.023	0.001	38.027	< 2e-16	389	0.389
	Log(theta)	1.702	0.126	13.462	< 2e-16		
		Zero-inflation model coefficients (binomial with logit link)					
	Intercept	-0.449	0.071	-6.336	2.36E-10	921	0.921
	Ancestors	-0.012	0.002	-6.537	6.27E-11	82	0.082
		Count model coefficients (negative binomial with log link)**					
Nobel Descendants	Intercept	-1.049	0.083	-12.680	< 2e-16	850	0.85
	Nobel Status	1.033	0.185	5.588	2.30E-08	0	0
	Descendants	0.002	0.000	17.193	< 2e-16	143	0.143
	Log(theta)	-1.497	0.059	-25.172	< 2e-16		
		Zero-inflation model coefficients (binomial with logit link)					
	Intercept	4.376	0.097	45.120	< 2e-16	997	0.997
	Descendants	-0.161	0.011	-14.610	< 2e-16	0	0
		Count model coefficients (negative binomial with log link)***					
Nobel M/GM	Intercept	-5.205	0.074	-70.656	< 2e-16	1000	1
	Nobel Status	2.354	0.242	9.708	< 2e-16	0	0
	M/GM	0.163	0.009	18.877	< 2e-16	0	0
	Log(theta)	-2.528	0.120	-21.124	< 2e-16		
		Zero-inflation model coefficients (binomial with logit link)					
	Intercept	-2.409	0.465	-5.181	2.21E-07	1000	1
	M/GM	0.048	0.008	6.263	3.77E-10	0	0
		Count model coefficients (negative binomial with log link)****					
Local Nobel Academic Family Members	Intercept	-1.714	0.016	-107.770	< 2e-16	0	0
	Nobel Status	1.860	0.098	18.950	< 2e-16	0	0
	Local Family	0.014	0.000	46.310	< 2e-16	964	0.964

	Predictor	Estimate	S.E.	t value	Pr(>|t|)	Number Random>Obs.	Adj. P Value
		Quasibinomial with logit link					
Local Nobel Heterogeneity	Intercept	-1.702	0.068	-25.093	< 2e-16	1000	1
	Nobel Status	0.273	0.146	1.867	0.062	347	0.347
	Nobel Family	0.072	0.019	3.874	0.0001	177	0.177

*Dispersion = 1.57; z = 32.15; $p < 0.0001$: ** Dispersion = 7.06; z = 10.45; $p < 0.0001$
Dispersion = 1.54; z = 5.78; $p < 0.0001$: * Dispersion = 2.16; z = 25.61; $p < 0.0001$
